# Treatment of Fever

**Published:** 1891-01

**Authors:** 


### TREATMENT OF FEVER.

For some years back the idea has prevailed that the great danger in fever is a high temperature, and the remedies at present most popular in the treatment of the various forms of febrile diseases, are known as anti-pyretics, among which are anti-pyrene, anti-fibrine, and a great variety of similar drugs. We have, from the first announcement of these remedies, opposed their use, for the reason that they have no hand in removing the causes of the disease for which they are administered. Professor Cantanni, of Naples, whose authority as an experienced and observing physician is second to no contemporary, has recently brought forward a very interesting theory respecting the relation of heat to fevers. It is not generally known that the high temperature connected with febrile disease is the result of the poisonous matters developed, but the germ causes of the disease. The cure of the disease necessitates the destruction of the germs. Professor Cantanni holds that the elevation of temperature is one of nature's methods of destroying the germs to which the fever is due, and that any medicinal agent, the administration of which has the effect to simply lower the temperature, is a direct damage, since it paralyzes the efforts of nature to antagonize the disease. This theory is one of great interest, and if generally adopted, will revolutionize the treatment of fevers. Dr. Cantanni recommends the use of water as the only safe and proper method of lowering the temperature in fever. The method of treating typhoid fever by means of baths, is obligatory in the French army. As the result, the mortality, which was 24 per cent. in 1865, had fallen to 11 per cent. in 1876, and to 9 per cent. in 1883.

For Sleeplessness.—Horsford's Acid Phosphate.—Dr. C. R. Dake, Belleville, Ill., says: "I have found it, *and it alone*, to be capable of producing a sweet and natural sleep in cases of insomnia from overwork of the brain, which so often occurs in active professional and business men."

